# Long COVID: a narrative review of the clinical aftermaths of COVID-19 with a focus on the putative pathophysiology and aspects of physical activity

**DOI:** 10.1093/oxfimm/iqac006

**Published:** 2022-09-16

**Authors:** Simon Haunhorst, Wilhelm Bloch, Heiko Wagner, Claudia Ellert, Karsten Krüger, Daniel C Vilser, Kathrin Finke, Philipp Reuken, Mathias W Pletz, Andreas Stallmach, Christian Puta

**Affiliations:** Department of Sports Medicine and Health Promotion, Friedrich-Schiller-University Jena, Jena, Germany; Department of Movement Science, University of Münster, Münster, Germany; Department for Molecular and Cellular Sports Medicine, Institute for Cardiovascular Research and Sports Medicine, German Sport University Cologne, Cologne, Germany; Department of Movement Science, University of Münster, Münster, Germany; Department for Vascular Surgery, Lahn-Dill Clinics Wetzlar, Wetzlar, Germany; Department of Exercise Physiology and Sports Therapy, Institute of Sports Science, Justus-Liebig-University Giessen, Giessen, Germany; Hospital for Pediatrics and Adolescent Medicine, Jena University Hospital, Jena, Germany; Department of Neurology, Jena University Hospital, Jena, Germany; Clinic for Internal Medicine IV (Gastroenterology, Hepatology and Infectious Diseases), Jena University Hospital, Jena, Germany; Institute for Infectious Diseases and Infection Control, Jena University Hospital, Jena, Germany; Clinic for Internal Medicine IV (Gastroenterology, Hepatology and Infectious Diseases), Jena University Hospital, Jena, Germany; Department of Sports Medicine and Health Promotion, Friedrich-Schiller-University Jena, Jena, Germany; Center for Interdisciplinary Prevention of Diseases related to Professional Activities

**Keywords:** Long COVID, post-acute sequelae, COVID-19, long-term symptoms, exercise, pathophysiology

## Abstract

The pandemic coronavirus disease 2019 (COVID-19) can cause multi-systemic symptoms that can persist beyond the acute symptomatic phase. The post-acute sequelae of COVID-19 (PASC), also referred to as Long COVID, describe the persistence of symptoms and/or long-term complications beyond four weeks from the onset of the acute symptoms and are estimated to affect at least 20% of the individuals infected with SARS-CoV-2 regardless of their acute disease severity. The multi-faceted clinical picture of Long COVID encompasses a plethora of undulating clinical manifestations impacting various body systems such as fatigue, headache, attention disorder, hair loss, and exercise intolerance. The physiological response to exercise testing is characterized by a reduced aerobic capacity, cardiocirculatory limitations, dysfunctional breathing patterns, and an impaired ability to extract and use oxygen. Still, to this day, the causative pathophysiological mechanisms of Long COVID remain to be elucidated, with long-term organ damage, immune system dysregulation, and endotheliopathy being among the hypotheses discussed. Likewise, there is still a paucity of treatment options and evidence-based strategies for the management of the symptoms. In sum, this review explores different aspects of Long COVID and maps the literature on what is known about its clinical manifestations, potential pathophysiological mechanisms, and treatment options.

**Introduction**

The emergence of the novel beta-coronavirus, termed severe acute respiratory syndrome coronavirus 2 (SARS-CoV-2), in late 2019 and its global spread in the following months has led to strained health resources around the world [1]. SARS-CoV-2 is a virus that is predominantly spread via respiratory droplets and aerosols [2] and as such causative of the pandemic coronavirus disease 2019 (COVID-19). Among the dominant acute symptoms of COVID-19 are fever, cough, and shortness of breath [3]. Beyond that, several multi-systemic complications have been linked to COVID-19, such as myocarditis, thrombotic events and acute kidney injury [4]. A hallmark feature of COVID-19 is endothelialitis resulting in microthrombi and angiogenesis that has been observed primarily in pulmonary capillaries but also in other organs [5]. As of July 2022, the number of people who have officially contracted SARS-CoV-2 has surpassed 540 million and, albeit being mostly associated with disease courses that do not require acute hospital care, approximately 1.8% of them have died because of COVID-19 (https://covid19.who.int). Yet, the burden of disease is not restricted to its case-mortality rate, as it has become evident that having survived COVID-19 is not equivalent to being recovered. It is now well recognized that a considerable number of patients diseased with COVID-19 experience symptoms that outlast the acute phase of the disease and persist for at least weeks or months. These post-acute sequelae of COVID-19 (PASC), also referred to as Long COVID, can be persistent or exacerbated manifestations of the acute symptoms, and new-onset symptoms [6]. Specifically, a broad range of clinical presentations, including fatigue, dyspnoea, and attention disorder is described that leave the affected patients struggling with the aftermath of COVID-19 every day, with a high percentage unable to exercise or complete tasks of daily living [7].

Nonetheless, the research is still in its infancy and to this day there is a paucity of knowledge regarding virtually all aspects of Long COVID. Many different pathophysiological mechanisms underlying Long COVID have been described and several principles for the treatment have been proposed. Additionally, there exists an uncertainty regarding how many people experience residual symptoms and if they might be part of distinct syndromes. Therefore, it is the objective of this review to map the existing literature on the classifications, clinical manifestations, potential pathophysiological mechanisms and to provide an evidence-based contextualization of the proposed strategies for the management of the PASC in the context of physical activity.

## Definition

*Nomenclature*

A range of different terms is used to describe the long-term symptoms of COVID-19 in the scientific literature and public media since a universally accepted nomenclature and definition has yet to evolve. Being described as one of the first diseases that has ever been characterized through social media platforms, the term "Long COVID" was coined by patient groups not long after COVID-19 turned into a global pandemic to bring attention to the lingering, non-resolving manifestations of the disease [8,9]. Subsequent scientific publications took this term up [10–12], extended it, or established divergent terms. Correspondingly, the terms "Long-haul COVID" [13], "Chronic COVID-19" [1], "Post-acute COVID-19" [14], "Post-COVID-19 syndrome" [15] or "Post-acute Sequelae of COVID-19 (PASC)" [16] are used to refer to remaining symptoms of an acute COVID-19 infection.

Reflecting the many different terms, multiple criteria have been used in studies to define from what time point residual symptoms should be regarded as aftermaths of COVID-19 [17–19]. According to a literature review by Martimbianco et al. [17] the time points used to assess symptoms defined as Long COVID ranged from three weeks after the acute infection to 24 weeks after hospital discharge among the studies included, indicating that different definitions might exist depending on the severity of the symptoms, or the patient group investigated. The currently most widely used nomenclature differentiates between the acute phase and the post-acute phase of COVID-19 based on data concerning the virus shedding. Since generally no replication-competent virus can be identified beyond three to four weeks after the onset of the symptoms [1,20], it has been suggested to define all symptoms that persist or newly appear after that time as "post-acute COVID-19" or "Long COVID" [1,14,21,22]. Further differentiations that were also adopted by multiple institutions were proposed to describe patients in different phases of symptom duration [1,15,18,23]. Accordingly, symptoms and abnormalities of COVID-19 that are present from weeks 4-12 following the acute illness are defined as "sub-acute" or "ongoing symptomatic COVID-19". Symptoms and abnormalities of COVID-19 that are present beyond twelve weeks of the acute illness and are not attributable to alternative diagnoses are termed "post-COVID syndrome" [1,15] or according to the latest clinical case definition by the WHO "post COVID-19 condition" [24] (Figure 1).

*Prevalence estimates*

Empirical findings suggest, there is great variance also in the prevalence estimates of Long COVID, ranging from 4.7% [25] to 89% [26] of the infected patients across different studies. These substantial differences between studies may be explained by several factors, such as the population and the follow-up period investigated, as well as by methodological challenges. The prevalence of Long COVID appears to be the highest among patients that required acute hospital care, although the acute disease severity cannot be relied on as an isolated predictor for the development of long-term symptoms and there may be a publication bias since there are far fewer studies investigating COVID-19 outpatients. Multiple cross-sectional studies of cohorts after hospital discharge report that the proportion of patients that experience Long COVID ranges from 52% to 89% between one and two months, from 46% to 68% around six months, and from 40% to 49% around 12 months post-discharge [26–31]. The largest longitudinal study of hospitalized patients to this day, including the first people admitted to the hospital in Wuhan, China, reported that 49% of the survivors experienced at least one residual symptom one year post-discharge [28].

On the other hand, the British National Institute for Health Research (NIHR) presumes that at least 20-30% of patients who were not admitted to the hospital experience at least one persistent symptom after one month [32]. Nevertheless, since the criteria for hospital admission might vary between different countries and many studies do not specify the level of care the patients received or the criteria used to define Long COVID, caution is advisable when interpreting and comparing the results of different studies [32]. Also, the fact that some studies investigated subjects with an assumed COVID-19 diagnosis and others only recruited subjects with PCR-confirmed diagnosis at times or in countries where widespread community testing was not available might additionally compromise the comparability of the study results [32]. It is furthermore reasonable to take the length of the follow-up into account when assessing the prevalence of Long COVID, as the persistence or occurrence of prolonged symptoms has been reported to change over time. A still not well-characterized proportion of patients experiences symptom relief after some time, resulting in lower prevalence estimates as time progresses. Multiple observational studies investigating self-reported symptoms describe a gradually decreasing number of patients reporting at least one residual symptom over several months after symptom onset [7,25,33,34]. Indeed, the Coronavirus Infection Study conducted by the British Office for National Statistics (ONS) found that around 20% of individuals testing positive for COVID-19 experienced at least one residual symptom after five weeks or more and 10% after twelve weeks or more [35].

*Disease burden*

Irrespective of the vagaries of the prevalence of Long COVID, what is clear is the high burden patients suffer in the wake of COVID-19. As ONS reported in July 2021, all measures of personal well-being, such as life satisfaction and happiness were worse among patients that experience long-term symptoms compared to those who had "Short COVID": 57% of patients with Long COVID said that it had affected their general well-being; 30% reported that it had compromised their ability to exercise and 39% their ability to work [36]. Moreover, at a community level, Briggs and Vassall [37] cautiously estimated that 30% of the global COVID-19 health burden may be attributable to acquired disabilities, not due to death. Especially in younger age groups, the burden in terms of lost years of healthy life due to chronic illness might be higher than due to death. Beyond that, the reduced work capacity might lead to long-term sickness absences with increasing socioeconomic costs [38].

## Clinical Manifestations

*Dominant symptoms*

Long COVID encompasses a multitude of clinical manifestations, affecting various body systems [1,39–42]. A study investigating an international cohort of 3762 individuals with long-term symptoms of COVID-19 identified 203 symptoms in ten organ systems, emphasizing the multi-faceted clinical picture of Long COVID [7]. Referring to the organ system in which the symptom manifested, they were described as systemic; neuropsychiatric; cardiovascular; dermatologic; gastrointestinal; musculoskeletal; immunologic/autoimmune; pulmonary/respiratory; reproductive/genitourinary/endocrine; or related to head, ear, eye, nose, throat [7]. Among the most reported symptoms are fatigue, headache, brain fog and dyspnoea [7,43–46]. But persistent gustatory and olfactory impairments are prevalent complaints, too, as the results of a recent German cohort study suggest [47]. A meta-analysis that aimed to estimate the prevalence of post-acute COVID-19 symptoms among 15 studies that assessed patients two weeks or more after initial symptom onset reported that the most common clinical manifestation was fatigue, giving a pooled prevalence of 58% (95% CI 42–73) among patients with Long COVID [45]. Headache (44%, 95% CI 13–78), attention disorder (27%, 95% CI 19–36), hair loss (25%, 95% CI 17–34), and dyspnoea (24%, 95% CI 14–36) followed in the list of the five most prevalent symptoms [45]. In accordance with the definitions of NICE, Iqbal et al. [44] characterized the prevalence of clinical features differentiating between the acute (3-12 weeks after symptom onset) and chronic post-COVID syndrome (beyond twelve weeks of symptom onset). The results of the meta-analysis including 30 studies with a follow-up period of at least 21 days suggests that the dominant symptoms in the weeks 3-12 after symptom onset were fatigue (37%, 95% CI 20-56), dyspnoea (35%, 95% CI 16-56), and anxiety (29%, 95% CI 19-40). During the chronic post-COVID stage, the pooled prevalence of fatigue and dyspnoea increased to 48% (95% CI 23-73) and 39% (95% CI 16-64), respectively [44] (Figure 1).

Another meta-analysis of studies that followed up symptoms of COVID-19 beyond two weeks from symptom onset, analysed the prevalence of post-acute symptoms separately for different timeframes. Similar to Iqbal and colleagues, they reported that at a follow-up of three months fatigue (35%, 95% CI 25-47), dyspnoea (26%, 95% CI 9-35), and myalgia (11%, 95% CI 7-18) were the most prevalent symptoms [48]. By contrast, the analysis of symptoms one month after symptom onset revealed that the dominant symptoms here were cough (19%, 95% CI 11-31), anosmia (17%, 95% CI 10-26), and ageusia (16%, 95% CI 9-27). As these symptoms are reminiscent of the dominant symptoms of an acute SARS-CoV-2 infection [3], it might be assumed that they are directly attributable to the acute infection and could be a reflection of a slightly delayed recovery. A considerable proportion of patients suffering from these lingering symptoms might consequently recover spontaneously over the following months, an assumption that gets supported by overall decreasing prevalence estimates [7,25,33]. On the contrary, symptoms that might reflect systemic homeostatic aberrations or extended autonomic system dysfunctions like fatigue, headache, myalgia or attention disorder might be the dominant symptoms beyond three months.

*Intra-individual characteristics*

The observation that the clinical manifestations of Long COVID are subject to considerable dynamics and undulating trajectories is not only illustrated by varying symptom prevalence. The episodic nature of Long COVID encompasses relapses, newly appearing symptoms and the feeling that symptoms are coming and going [7,25,49,50]. 43% of patients treated in a French outpatient clinic with persisting or remitting symptoms of COVID-19 reported that symptom-free intervals of a few days or hours alternated with sudden relapses. Newly appearing symptoms, that had not been present in the acute phase, were described by 76% of the investigated patients [49]. Similarly, Ziauddeen et al. [50] described that patients in an online survey experienced fluctuating (59%) and relapsing (16%) symptoms. Taking the intra-individual differences of Long COVID into account, Fernández-de-las-Peñas et al. [6] proposed a model which classifies the symptoms of patients as exacerbated, delayed-onset, or persistent.

In the aforementioned study by Davis et al. [7] 86% of the participants reported relapses. These relapses occurred either irregularly or as a reaction to specific triggers such as physical or mental exertion, alcohol, menstruation or stress. Beyond that, the analysis of the course of 66 symptoms over the period of seven months revealed specific patterns in the progression of the symptoms over time. While the appearance of some symptoms was more likely in the early phase, others were more likely to appear in the later months and become chronic [7]. The authors grouped symptoms with similarly shaped time courses into three clusters to illustrate the intra-individual undulating character of Long COVID. Symptoms in cluster one were more likely to appear in the early phase, e.g., in the first three weeks after the acute phase and encompassed for example loss of appetite, sore throat, dry cough, fever, or a runny nose. Symptoms in cluster three (dermatologic manifestations, new allergies, brain fog, memory issues) were more likely to appear during later stages, whereas the likelihood of symptoms in cluster two (tachycardia, fainting, muscle aches, headache, breathing difficulties, fatigue) remained constant with a slight plateau around the second month after symptom onset [7].

*Inter-individual characteristics*

In addition to undulating courses of the symptoms, Long COVID is also characterized by a large inter-individual heterogeneity. Both the number and the type of symptoms experienced have been reported to vary between patients [51]. While some exhibit one residual symptom, most patients suffer from more than two and up to ten or more symptoms at the same time [7,39,48,51]. Reflecting on the heterogenous clinical presentations, some studies attempted to classify the symptoms and to identify common patterns among them. Venturelli et al. [52] argued that it might be reasonable to separate three distinct syndromes, especially in a sample that involves many subjects with a history of hospitalization:

1) post-viral chronic fatigue syndrome;

2) post-critical-illness syndrome; and

3) post-traumatic stress disorder.

Including non-hospitalized patients at a minimum of four weeks after initial symptom onset, Sahanic et al. [53] proposed three phenotypes of Long COVID:

1) the anosmia/hyposmia phenotype including taste and smell disorders;

2) the fatigue phenotype including fatigue, tiredness, concentration and memory deficits and

3) the multi-organ phenotype including pulmonary, gastrointestinal, neuro-cognitive and cardiovascular disorders.

Similarly, as a part of the REACT-2 study, Whitaker et al. [54] reported the existence of two (overlapping) symptom profiles twelve weeks after symptom onset:

1) the tiredness cluster encompassing high prevalence of tiredness, muscle aches, sleep difficulties and

2) the respiratory cluster encompassing high prevalence of shortness of breath, tight chest, chest pain (Figure 1).

Notably, the respiratory cluster contained around three times fewer patients than the tiredness cluster, the share of patients reporting a severe acute disease was however higher in the respiratory cluster (44% vs. 27%) [54]. A clear-cut differentiation between clusters is not possible and overlapping symptom presentations possible. Beyond the symptom-based differentiation, Long COVID might also encompass several non-exclusive sub-diagnoses, such as postural orthostatic tachycardia syndrome (POTS) [55–57], mast cell activation syndrome (MCAS) [58,59], small fibre neuropathy (SFN) [60] or dysautonomia [61,62]. Yet, there is still no universally agreed classification of distinctly different phenotypes or syndromes under the umbrella of Long COVID.

*Overlap with other post-infectious sequelae*

The clinical presentations of Long COVID are not entirely unknown but share several similarities with other post-infectious syndromes and maladaptive conditions. For instance, a review that compared the symptomatology of Long COVID and Myalgic Encephalomyelitis/Chronic Fatigue Syndrome (ME/CFS) concluded that out of 29 known symptoms of ME/CFS, 25 were also present in Long COVID patient cohorts [63]. Indeed, it has been documented that a considerable number of patients with ME/CFS report that their disease was preceded by infectious-like symptoms, albeit an exact estimation of their proportion remains elusive [64,65]. Komaroff and Lipkin [66] reported that ME/CFS-like fatigue syndromes are documented after infections with herpes viruses (Epstein-Barr virus, human cytomegalovirus, human herpesviruses 6A and 6B), enteroviruses, Ebola virus, West Nile virus, coronaviruses and several bacterial strains like Coxiella or Mycoplasma pneumoniae. Post-acute sequelae resembling the symptomatology of Long COVID are furthermore documented after infections with measles virus, adenovirus, influenza virus, zika virus, tick-borne encephalitis virus and many other infectious agents [16,67].

*Exercise intolerance*

Many patients with Long COVID report that their ability to exercise is negatively affected by a diminished physical capacity and an impaired ability to recover from physically or mentally exhausting stress [36]. In a prospective cohort study, a reduced exercise capacity was the most frequently reported symptom one year after symptom onset, affecting more than half of the investigated subjects [68]. A pronounced exercise intolerance and a condition referred to as post-exertional malaise (PEM) are hallmark symptoms of Long COVID that often go along with fatigue [7,69]. PEM is characterized by a prolonged recovery and deterioration of one or multiple symptoms immediately or 24 to 72 hours following physical activities that are not alleviated by rest or sleep [69]. Most patients with PEM investigated in a survey indicated that their symptom exacerbation lasted at least a few days

[7]. Consequently, many patients with PEM experience not only substantial difficulties to exercise but also completing activities of daily living.

In addition to a pathological recovery from exertion, patients with Long COVID exhibit acute restraints of cardiopulmonary function such as dyspnoea, palpitations, or tachycardia during strenuous activities. Moreover, as the results of several studies employing cardiopulmonary exercise testing (CPET) suggest, patients recovering from COVID-19 tend to have a reduced aerobic capacity and to attain the anaerobic threshold early, regardless of their acute disease severity [70–77] (Table 1). The aerobic capacity is often described as the maximal/peak amount of oxygen a subject can use per kilogram and unit of time ($VO_{2max}$ / $VO_{2peak}$). $VO_{2max}$ and $VO_{2peak}$ are key determinants of endurance exercise capacity and are the most common measures of maximal functional aerobic capacity. Its assessment allows a conclusion to be drawn about the proportion of aerobic and anaerobic energy production during physical activity at different intensities. The switch towards energetically less efficient anaerobic metabolic pathways ultimately limits the amount of energy that can be provided for cellular processes. With only one study finding no significant differences in relevant CPET variables between the PASC and the control group [78], the finding of a reduced aerobic capacity in a variable proportion of patients with Long COVID is consistent across many studies. Still, the mechanisms underlying the diminished exercise capacity are not described as unequivocally, as several physiological factors need to be considered.

A central factor that has been reported to contribute to the exercise limitation is the inability to adequately adjust the cardiac output to the exercise demand. Szekely and colleagues [76] for example reported that 75% of the investigated non-hospitalized patients demonstrated chronotropic incompetence at a mean follow-up of three months. Furthermore, at peak exercise, the stroke volume, left ventricular ejection fraction, and left ventricular end-diastolic volume were significantly lower in the post-COVID group than in the control group. The higher arterio-venous oxygen difference in the post-COVID group, indicative of an upregulated oxygen extraction, could ultimately not compensate for the reduced cardiac output, leading to a lower $VO_{2peak}$ [76]. Cardiocirculatory limitations such as a decreased heart rate reserve at peak exercise were also reported by other studies [70,74,77]. In addition to that, peripheral restraints such as the inability to extract oxygen from the blood [75] or an impairment to use oxygen and clear lactate secondary to mitochondrial reprogramming [71,77] have been reported. To investigate the latter, de Boer et al. [77] assessed several surrogates of mitochondrial function during Ramp protocol in 50 subjects with persistent symptoms at a mean follow-up of six months after symptom onset. At peak exercise, subjects with Long COVID showed significantly lower levels of ß-oxidation of fatty acids and higher blood lactate concentrations compared to control subjects. This finding could serve as an indication for a premature switch to anaerobic glycolysis and a loss of mitochondrial flexibility in patients with lingering symptoms of COVID-19 [77].

Finally, some patients exhibited dysfunctional breathing patterns and a ventilatory inefficiency marked by greater minute ventilation to carbon dioxide output ratio ($VE/VCO_2$), hypocapnia, and respiratory alkalosis during peak exercise [72,73,75,79]. Accordingly, the impaired ability to get oxygen into the bloodstream, to deliver it, or to use it in the periphery and resulting tissue hypoxia could not only be a mechanism underlying the reduced exercise capacity but also for the prolonged recovery, albeit the recovery was not specifically investigated in the referenced publications.

**Potential pathophysiological mechanisms**

Just as Long COVID's clinical manifestations are multifaceted, so are its underlying pathophysiological mechanisms that are currently discussed. It bears noting that at the moment the understanding of the mechanisms involved in the development of Long COVID is still evolving and that the explanatory attempts have to be regarded as theories rather than evidence. Nalbandian et al. [1] proposed that the potential mechanisms include virus-specific pathophysiologic changes, immunologic aberrations, and inflammatory damage in response to the acute infection and expected sequelae of post-critical illness. Other reviews [80,81] added that certain organ-specific symptoms might be a result of acute viral or inflammatory damage to specific organs. Symptoms that manifest rather systemically might on the other hand be a product of a subsequent disruption of homeostatic systems. Accordingly, metabolic dysregulation, autoimmunity, renin-angiotensin-aldosterone-system, and endothelial dysfunction as well as microbiome-virome dysbiosis might contribute to the pathophysiology of Long COVID (Figure 2). It can be assumed that the proposed mechanisms are not exclusive. Given the heterogeneity of the sequelae, a combination of pathophysiological mechanisms mediated by a set of individual risk factors such as biological sex [82–84], acute disease severity [85,86], and symptoms in the acute phase [25,87–89] might cause the lingering symptoms [90].

*Sequelae of direct viral toxicity*

Based on findings that a considerable number of patients with lingering symptoms shows signs of persistent organ damage or impaired function [39,73,91], it has been theorized that lasting structural damage due to the cytopathic effects of viral replication contributes to Long COVID [19,66]. SARS-CoV-2 infects host cells through binding of its spike protein's receptor-binding domain to the angiotensin-converting enzyme 2 (ACE2) receptor [2,92]. As part of its replicative cycle, SARS-CoV-2 induces death and injury to infected cells, a process that compromises organ infrastructure [2]. As a respiratory virus, it is known to be primarily transmitted via respiratory droplets [2,93]. Entering the body through the upper respiratory tract, SARS-CoV-2 consequently infects nasopharyngeal or alveolar epithelial cells and type 2 pneumocytes that express high levels of ACE2 [93–95]. Pathological or delayed tissue remodelling might in consequence, especially in the early phase, contribute to the frequently observed pulmonary manifestations of Long COVID, such as breathlessness and cough [92]. Correspondingly, a study by Raman et al. [73] that investigated subjects 2-3 months after hospital discharge found that 60% of them showed abnormalities in the lungs. Likewise, several other studies described substantial proportions of patients with fibrotic lung damage regardless of their acute disease severity [96,97]. Among the resulting functional impairments are a limited alveolar gas exchange and decreased diffusion capacity [98–102] that might lead to a shortage of oxygen, especially during exhausting activities. A systematic review and meta-analysis of studies with a mean or median follow-up of at least three months reported that 39% (95% CI 24–55) of the included patients had abnormal pulmonary function tests and 31% (95% CI 24–38) an impaired lung diffusion capacity [103]. Combined with a decreased ability of muscles and organs to use oxygen [75] and low levels of haemoglobin [104], a low blood oxygen saturation might further trigger hypoxic organ damage [105].

Functional and structural organ impairments are, however, not only documented for the respiratory organs but also for other tissues expressing ACE2. It has been theorized that several routes allow the virus to spread into other tissues, causing the previously mentioned multi-organ involvement [66,105]. Invasion through the neuronal-axonal route has been assumed to lead to damage to olfactory, neuronal and surrounding support cells resulting in a decreased ability to smell [8,106]. Viral spread through the hematogenous route and ACE2 expression in heart, pancreas, kidneys, intestines, brain, and vasculature could further explain conditions like heart failure, β-cell dysfunction, acute kidney injury, or endothelial dysfunction [18,93].

*Acute and chronic immune system dysregulation*

Besides the lasting effects of direct viral toxicity, it has been assumed that an aberrant immune response might contribute to non-resolving or newly appearing symptoms by inducing acute, long-lasting inflammatory damage and/or a chronic inflammatory profile [19,90].

The recognition of damage-associated molecular patterns by resident macrophages and epithelial cells during the acute illness triggers a robust innate immune response that can cause widespread collateral damage through excessive immune cell infiltration and overproduction of pro-inflammatory cytokines [2]. Correspondingly, the acute illness of severely ill patients, characterized by a dysregulated pro-inflammatory feedback loop with a surge in circulating cytokines, has previously been described as a cytokine release syndrome [2,107]. Tissue damage induced by the overreaching immune response could outlast the acute symptomatic phase and lead to residual functional organ impairments. The results from a German cohort study, for example, investigating patients at a median follow-up of 71 days, revealed that 78% of them had cardiovascular pathologies. Moreover, in 60% of the patients the cardiac MRI revealed ongoing myocardial inflammation [91]. Likewise, another study including female health care workers reported an isolated myocarditis in 26% at a mean follow-up ten weeks after infection [108]. Yet studies investigating different populations found much lower incidences of myocarditis, with 2.3% in a cohort of athletes and 0.08% in a cohort of adolescents [109,110]. Beyond that, a review by Guizani et al. [92] proposed that the infiltration of immune cells and release of cytokines trigger the activation of matrix metalloproteinases. The interplay of several mediators leads to the collapse and pathological remodelling of the lungs' extracellular matrix, favouring fibrotic adaptations.

In addition to acute immune system aberrations, it seems likely that a chronic inflammatory state exists through the post-acute phase [18,19,67]. The general immune profile of patients recovering from an acute COVID-19 infection, regardless of whether they experience residual symptoms or not, shows an up-regulation of a multitude of immunological signalling molecules associated with inflammation, vascular injury or immune cell differentiation [111–116]. Montefusco et al. [113] for instance described that patients recovering from COVID-19 had significantly increased levels of granulocyte-colony stimulating factor (G-CSF), macrophage inflammatory protein-1 beta (MIP-1$\beta$), tumor necrosis factor (TNF) and interleukin (IL)-1$\beta$, IL-4, IL-6, IL-7, IL-8, IL-10 and IL-13 as compared to healthy controls two months after index infection. Studies specifically characterizing the immune signature of patients with Long COVID described an elevated erythrocyte sedimentation rate, CRP, TNF-$\alpha$, IL-4 and IL-6 level [104,117,118]. Adding to this body of literature, it was demonstrated that eight months after the index infection, an increased concentration of IL-1$\beta$, IL-6 and TNF-$\alpha$ showed a significant correlation with persistent symptoms [119] and that a combination of inflammatory markers including interferon (IFN)-$\beta$ and IL-6 had a prognostic accuracy for Long COVID of 79-82% [120]. Accordingly, the contribution of a systemic inflammatory state and neuroinflammation have been documented for the pathogenesis of depressive disorders and other neurocognitive symptoms following COVID-19 [121–124]. Moreover, it is currently assumed that the dysregulated immune system activity and persistent inflammatory signalling contribute to the pathophysiology of many Long COVID symptoms by eventually disrupting several homeostatic systems [18,67].

*Metabolic dysregulation*

Similar to ME/CFS, a dysregulated metabolic state has been reported to be among the frequently observable clinical manifestations of Long COVID on a molecular level [80,104,113,125]. Specifically, an Italian study investigating 551 recovered patients two months after the onset of COVID-19 discovered that approximately half of them were hyperglycaemic, although no one had a history of glycaemic abnormalities [113]. Also, the homeostatic model assessment of insulin resistance (HOMA-IR) was significantly higher post-COVID-19 compared to controls and displayed a significant correlation with the inflammatory score. Correspondingly, the authors hypothesized that in a subset of patients, the excessive release and persistent presence of cytokines might trigger the onset of these metabolic alterations by affecting insulin resistance and $\beta$-cell function [113]. In addition to that, it has been documented that hypometabolism, mediated by a mitochondrial dysfunction, is a common feature in patients with Long COVID [80]. Mitochondrial hijacking by SARS-CoV-2 for the purpose of replication as well as an inflammatory state might divert the energetic capacities of infected and non-infected cells [80,125]. Moreover, several publications investigating the brains of patients with lingering symptoms using positron emission tomography detected a decreased metabolism in the frontoparietal and temporal lobes [126–128]. These hypometabolic patterns discriminated patients with Long COVID significantly from healthy controls and were significantly correlated with functional complaints such as cognitive impairment, fatigue, and insomnia [126,128]. A hypometabolic syndrome, the viral-induced shift towards glycolysis and inability to generate energy from multiple sources has previously been documented in patients with ME/CFS, as well [129]. It is therefore conceivable that an energy dys-homeostasis due to an impairment of metabolic control is associated with musculoskeletal, neuropsychiatric, and cognitive sequelae of COVID-19.

*Endothelial dysfunction*

Vascular events such as coagulation issues and microvascular injury have been described as being common complications of acute COVID-19 [94,95]. The apoptosis of endothelial cells induced by direct infection by SARS-CoV-2 as well as the cytokine-mediated platelet activation and leukocyte adhesion disturbs the vascular homeostasis [111,130]. Furthermore, the binding and subsequent down-regulation of ACE2 leads to an accumulation of angiotensin II and reactive oxygen species and disturbs the nitric oxide (NO) production [80]. Combined, these processes impose damage to the endothelium and the vascular system by leading to a redox imbalance, increasing oxidative stress, eliciting mitochondrial dysfunction and fuelling inflammation [80,131,132]. It has been suggested that a persistent endothelial dysfunction, maintained by a residual immune activation, is present in patients with Long COVID and contributes to its symptomatology [130,133]. Chioh et al. [111] detected significantly elevated levels of circulating endothelial cells (CEC), which serve as a biomarker of vascular injury, in COVID-19 convalescents compared to healthy controls. According to the authors, the fact that CEC correlated positively with inflammatory cytokines could implicate an endotheliopathy with an inflammatory aetiology that outlasts the acute phase [111]. Likewise, indicators for a persistent endothelial dysfunction such as a low nitrite/nitrate ratio, and decreased endothelium-dependent flow-mediated dilation (FMD) have been consistently reported among COVID-19 convalescents [130,134,135]. A prospective observational study investigating the vascular adaptions to short-term occlusion-induced ischemia found that Long COVID patients exhibited significantly reduced endothelium-mediated dilation of peripheral arteries compared to controls and patients in the acute COVID-19 stage, suggesting a possibly impaired ability to appropriately adjust the vascular tone as a chronic complication of COVID-19 [136]. Likewise, Nandadeva et al. documented a decreased brachial artery FMD in young adults with persistent symptoms compared to healthy and asymptomatic controls at least four weeks after COVID-19 diagnosis [137]. A maladaptive arterial dilation was also significantly associated with fatigue, chest pain, and neuro-cognitive deficits in a study including 618 Long COVID patients [138]. Furthermore, the increase of peak thrombin, shorter thrombin lag times, elevated D-dimer levels, and the finding of fibrinolysis-resistant microclots suggest a persistent pro-thrombotic state and increased risk of thromboembolic events in Long COVID patients [112,134,139,140]. Altogether, it is assumed that pathologies arising from a dysfunctional endothelium contribute significantly to the development and maintenance of Long COVID.

*Persistence and reactivation of harboured pathogens*

According to a comprehensive review of biological factors underlying Long COVID, the immune system dysregulation in the wake of a COVID-19 infection opens the opportunity for a reactivation of previously acquired and since harboured viruses [16]. Among viruses that have been found in human tissue reservoirs are for example the Epstein-Barr virus, herpes simplex virus or varicella zoster virus [16]. Together with bacteria and fungi, latent viruses enter a meta-stable microbiome-virome balance that is normally checked by a competent immune system, specifically by interferons [16,93]. As coronaviruses effectively suppress the host's interferon response [141], latent viruses can be reactivated, possibly causing virus-specific symptoms, further immune activation or conditions resembling acute sickness behaviour. A study by Gold et al. [142] revealed that two-thirds of the investigated patients with Long COVID showed signs of an Epstein-Barr virus reactivation based on antibody titres. There is also strong evidence suggesting that SARS-CoV-2 can persist in gastrointestinal, hepatic, or lung tissues [143–145]. In line with this, it has for example been documented that SARS-CoV-2 was reactivated in an immune-compromised host that was readmitted to the hospital with reappearing symptoms after a symptom-free interval of four months. Sequencing analysis and comparison with the first sample revealed that both isolates belonged to the same strain that had persisted and evolved in the host [146].

*Autoimmunity*

A frequently described sequela of SARS-CoV-2 infection and the subsequent immune system dysregulation is the generation of autoantibodies [147]. The hyperactivation of the immune system accompanied by molecular mimicry between SARS-CoV-2 antigen and host protein appear to be the main underlying mechanisms of post-infectious autoimmunity [16,147]. Correspondingly, a wide range of autoantibodies specific to G-protein coupled receptors (GPCR), chemokines, cytokines or tissues, and cell structures such as vascular cells, platelets, and connective tissue has been detected in acutely ill [148], convalescent [149], and Long COVID patients [150]. An impeded catecholamine and acetylcholine signalling could consequently cause autonomic dysfunctions including tachycardia, disturbances in the control of the vascular tone and coagulation as well as an impaired ability to contain inflammation [151]. In one case report, a DNA aptamer drug neutralized autoantibodies targeting GPCR, resulting in a significant improvement of retinal capillary microcirculation and disappearance of Long COVID symptoms. The authors hypothesized that functional vasoactive GPCR-autoantibodies contribute to the impairment of the microcirculation, not only in the retina but in the entire body [152]. Moreover, the attack of host cells by autoantibodies might further compromise organ infrastructure, reminiscent of other diseases with an autoimmune aetiology such as rheumatoid arthritis, connective tissue disease or fibromyalgia [153]. Correspondingly, Seeßle et al. [68] demonstrated that patients found to have antinuclear antibody titres in excess of 1:160 had a significantly higher frequency of Long COVID symptoms one year after the infection than those who had titres below this threshold.

*Morpho-functional changes of blood cells*

Acute COVID-19 can lead to changes in blood cell count, morphology, and function, affecting leukocytes, erythrocytes, and thrombocytes [154–156]. In the acute phase, these changes can play a critical role in the development of severe disease. Hyperactive neutrophils (increased calprotectin), pronounced lymphocytopenia, or NK-cell exhaustion are for example associated with severe COVID-19 [154,155]. Likewise, it has been documented that COVID-19 induces a decrease in haemoglobin levels, an increase in red blood cell distribution width, and irreversible damage to the erythrocyte membrane structure [157–159].

Persistent morpho-functional changes have been hypothesized to contribute to the pathophysiology of Long COVID [158]. Yet, there is a paucity of evidence investigating such changes during COVID-19 convalescence, let alone in Long COVID. Nonetheless, it is conceivable that acute erythrocyte alterations might outlast the acute illness, with one of the reasons being that red blood cells have an average life span of around 120 days, indicating that damaged cells can remain in the circulation for up to three months before being replaced [158]. Indeed, a study investigating the physical phenotype of several blood cells using real-time deformability cytometry reported decrements in erythrocyte size and deformability in subjects with COVID-19 that were still present even at a median of 7.1 months after hospital discharge [160]. In addition to that, significantly low levels of haemoglobin have been documented in patients with Long COVID [104]. Collectively, impairments in erythrocyte function might affect peripheral oxygen homeostasis, with implications especially for physical activities with increased oxygen demand [161]. Beyond that, morpho-functional changes in immune cells have been reported throughout the recovery period. Several studies have for example documented signs of T-cell exhaustion in patients with Long COVID that were interpreted as an indication for SARS-CoV-2 persistence or bystander activation [120,162]. These include increased expression of the activation and exhaustion markers PD-1 and TIM-3 in memory CD4+ and CD8+ T-cells cells for up to 8 months [120,163]. At the same time, compared to controls, subjects with Long COVID exhibited significantly decreased levels of naïve CD4+ T-cells [163,164].

*Post-intensive care syndrome*

Many patients with Long COVID were admitted to the hospital during their acute symptomatic phase, and a significant proportion required intensive care and life-saving measures. It has been documented that their risk of experiencing residual symptoms of COVID-19 is higher compared to individuals with a mild disease course [165]. As mentioned previously, the proportion of hospitalized patients that went on to have Long COVID around two months after discharge ranges from 52% to 89% [26,27,29,31]. A study that stratified the prevalence of residual symptoms based on the hospital care the patients received during acute illness provides further insight into how disease severity influences the persistence of symptoms. Accordingly, six months post-discharge, the proportion of patients with at least one persistent symptom was considerably higher among those who required mechanical ventilation compared to those who required supplemental oxygen, and those who required no supplemental oxygen (84% vs. 66%, and 69%, respectively) [28]. At a follow-up of 12 months, this difference was less prominent (52% vs. 49%, and 47%, respectively) [28].

In addition to the severe impact of the virus itself and the subsequent inflammatory response, many hospitalized patients had to battle the complications of oxygen shortage, prolonged bed confinement, mechanical ventilation, and coma. The inability to fully recover from the critical illness has been termed post-intensive care syndrome and encompasses symptoms such as memory deficits, depression, fatigue or dyspnoea that resemble Long COVID [19,166]. Stanculescu et al. [167] postulated that a circle consisting of a cytokine-depressed thyroid hormone function, leading to oxidative stress, which stimulates inflammatory cytokine production together with other endocrine dysfunction contributes to the prolonged recovery after intensive care.

**Symptom management and return to physical activity**

Reflecting the paucity of knowledge regarding most aspects of Long COVID, there is still no evidence-based or even commonly agreed upon strategy for the treatment of the variety of affected patients. A recent review reported that at the time of submission, fewer than 15 clinical trials had been registered to investigate potential treatments for Long COVID, of which the majority aimed to address drug treatments [51]. To date, there exist no controlled trials that would prove the efficiency and safety of a pharmaceutical drug that would significantly improve certain symptoms of Long COVID [19]. The use of anti-inflammatory steroids and anti-coagulants has been suggested by some authors for the treatment of specific symptoms, such as fever, thromboembolism or skin lesions [1,14,168]. These suggestions are, however, based on personal clinical evidence and experiences with comparable clinical presentations. The utility of medications for a wide range of patients still needs to be evaluated. For that reason, currently applied rehabilitation approaches are oriented towards the management of individual symptoms. Yet, the evidence here as well is sparse and relies mostly on experiences that were made in the context of other post-viral syndromes or with purportedly comparable conditions. Most of the existing observational studies [169–172] investigating rehabilitation approaches recruited patients recovering from COVID-19 in general and are hence hardly representative of the complex needs of patients with Long COVID. Likewise, many of the available editorials only address the rehabilitation post-COVID-19 but not of patients with lingering symptoms. Therefore, clinicians must refer mostly to consensus statements, guidelines, and experts' opinions in pursuit of choosing appropriate management strategies for Long COVID. According to Brown and O'Brien [173] and the Briefing Paper of World Physiotherapy [69], the rehabilitation should be centred around the individual's disabilities and goals and should focus on their function. Therefore, a holistic and multidisciplinary rehabilitation that takes the patients' comorbidities, mental health, and social needs into account has been suggested [14,21,174].

Beyond that, the current literature focuses on two roughly distinctive patient groups that differ with regard to the complexity of their clinical presentation and the resulting rehabilitation needs. The first group consists of patients deconditioned after a hospital stay and/or with discernible organ-specific sequelae, while the second group presents a complex clinical picture with the derailment of several autonomic systems and systemic symptoms such as fatigue, arthralgia, myalgia, or mood disorders. Correspondingly, several publications discuss principles and strategies for the management of symptoms that are attributable to a specific organ system. The management of commonly reported pulmonary sequelae could be, dependent on the specific pathology, comprised of aerobic exercises, breathing control exercises, relaxation techniques, or prescription of oral steroids [14,21,175–177]. In addition to that, specific recommendations exist for the management of cardiovascular, haematologic, endocrine, gastrointestinal, neurological, hepatic or renal sequelae [1,14,21,178], the description of which is beyond the scope of this review.

As the rehabilitation should be focused on function and individual impairments, physical activity is widely considered in the management of Long COVID. For many patients, the inability to exercise or complete tasks of everyday living represents a major disability [179]. Furthermore, a review by Jimeno-Almazán et al. [51], for instance, points out the potential benefits of exercise on the various body systems affected by Long COVID. As it has been shown with other pathologies similar to Long COVID, exercise might favour symptom resolution by modulating pain and mood, stimulating brain plasticity, or promoting mitochondrial biogenesis [51]. In addition to that, the immunomodulatory capability of exercise has been stressed, namely by the mobilization of effector cells and promotion of an anti-inflammatory cytokine profile [51,168]. Indeed, a case study of an otherwise healthy man has shown that cycling exercise mobilizes SARS-CoV-2 specific T-cells and raises the level of neutralizing antibodies in an intensity-dependent fashion, suggesting the possibility of an exercise-enhanced clearance of persistent virus [180]. However, in order to effectively combat viral reservoirs, lymphocytes need to be redeployed from the blood to the tissue compartment, a process that requires a certain sympathoadrenal activation. It remains to be elucidated if Long COVID patients are physically capable of reaching an exercise intensity threshold that is sufficient to cause this activation.

For the purpose of the patients' safety, specific criteria have been defined that should be met before resuming physical activities after COVID-19. These criteria might vary depending on the level and intensity the patient seeks to return to. As a general recommendation for patients discharged from the hospital, Demeco et al. [169] proposed that they should not resume physical activities if they have 1) a resting heart rate above 100 beats per minute; 2) a blood pressure below 90/60 mmHg or above 140/90 mmHg or 3) a blood oxygen saturation equal to or below 95%. A consensus statement published by Barker-Davies et al. [21] adds that patients experiencing a severe sore throat, body aches, shortness of breath, general fatigue, chest pain, cough or fever should avoid exercise (≥3 metabolic equivalents of task) for between two and three weeks after cessation of the symptoms. Furthermore, it has been recommended to exclude severe complications of COVID-19, such as myocarditis or pneumonia [181,182]. Subsequently, it has been suggested to follow rehabilitation protocols with cautious increase of load, albeit there is no specific framework available yet.

In addition, the promotion of physical activity might potentially be harmful for a significant number of patients that are either deemed unable to exercise due to severe complications of COVID-19 or exhibit complex symptom presentations such as fatigue or autonomic dysfunctions. As discussed previously, relapses and symptom exacerbations in response to physical exertion were reported by 86% of the participant of an online survey [7]. Reflecting the undulating nature of the symptoms, the advice is to screen the patient for post-exertional symptom exacerbations, including orthostatic intolerance, intermittent headache or debilitating fatigue before administering fixed rehabilitation protocols [183]. Beyond this, it is becoming apparent that patients with Long COVID can present with a loss of oxygen transport pathway integrity, mitochondrial capacity and ventilatory efficiency that may acutely impair exercise tolerance and ability to recover [71,72,75]. Thus, when constructing a rehabilitation programme, the type of exercise, its intensity and its duration will need to be tailored to a patient's exercise limitation. In keeping with that, the goal to employ a symptom-guided rehabilitation program that uses light activities to restore the patients' previous level of activity and to improve the quality of life has been proposed, rather than building strength or endurance [184]. Correspondingly, several publications promote the use of energy conservation strategies, such as pacing and working within individual limits for the management of a fatigue-dominant symptomatology [184,185]. Important principles in the context of pacing strategies are to avoid overexertion, allow adequate recovery periods, divide activities of daily living into smaller tasks that can be spread out over the day, and to rest before symptoms arise [185]. Additionally, a consensus statement on the treatment of fatigue in Long COVID patients recommends slowly advancing activities as tolerated and reducing them to the previously tolerated level when symptoms worsen [184].

**Conclusions**

COVID-19 is a disease that is associated with long-term symptoms and negative health outcomes that also affect individuals in good general health without any notable comorbidities before infection with SARS-CoV-2. Thus, with a rising number of people being infected and many that have already been infected, PASC will place a high burden on patients and on health resources for years to come. This review of the current literature illustrates the many conundrums surrounding Long COVID, its multi-faceted clinical manifestations, and potential causative pathophysiological mechanisms. Given the enormous and growing scale of this condition, a more thorough understanding of the pathogenesis is urgently needed. Additionally, research efforts should be invested in exploring treatment options, public care models and looming questions such as paediatric Long COVID, the influence of new variants, and breakthrough infections.

**Conflict of interest**

The authors declare that there is no conflict of interest associated with the publication of this review.

**Data availability statement**

No new data were generated or analysed in support of this research.

## Figures

**Figure 1:** Time course of Long COVID, dominant symptoms over time and proposed clinical phenotypes (Created with BioRender.com).

**Figure 2:** Putative pathophysiological mechanisms underlying Long COVID and associated clinical findings. GPCR: G-protein coupled receptors (Created with BioRender.com)

## Tables

**Table 1:** Overview of studies that investigated the physical capacity and exercise tolerance of patients recovering from COVID-19, employing cardiopulmonary exercise testing (CPET). AaDO2: alveolar-arterial oxygen difference; bpm: beats per minute; FATox: fat oxidation; HR: heart rate; HRR: heart rate reserve; mmol/l: millimoles per liter; NR: not reported; pred.: predicted; VE/VCO2: minute ventilation/carbon dioxide production ratio

| Reference | Location | Observation group at inclusion | | | Proportion of patients treated in hospital | CPET Protocol | Control group | Main findings |
|---|---|---|---|---|---|---|---|---|
| | | N | Follow-up | Residual symptoms | | | | |
| Alba et al. [78] | USA | 18 | 258 days (mean) | Dyspnea, exercise intolerance | 33% | Maximal effort on a cycle ergometer | 18 matched uninfected subjects with unexplained dyspnea and/or exercise intolerance | No difference in CPET variables (e.g., $VO_{2peak}$, peak workload, VE/$VCO_2$ slope) between groups, except for a higher $HR_{peak}$ in PASC cohort (p=0.02) |
| Clavario et al. [70] | Italy | 200 | 3 months | Fatigue, dyspnea, chest pain | 100% | Incremental, symptom-limited on a cycle ergometer | - | Reduced $VO_{2peak}$ (< 85% pred.) in 50% |
| De Boer et al. [77] | USA | 50 | 6 months (mean) | Dyspnea on exertion, chest pain | 10% | Maximal effort Ramp protocol on a cycle ergometer | Subjects from previously published cohorts of patients with metabolic syndrome and moderately active individuals | Reduced $VO_{2max}$ in 32% (<84% pred., of which 56% with HRR < 15bpm and 63% with low $O_2$ pulse at peak exercise); Higher mean lactate and lower FATox compared to controls (p<0.05) |
| Mohr et al. [71] | Germany | 10 | 115 days (mean) | Dyspnea | 60% | NR | - | Gap between preserved mean work rate (94% pred.) and reduced $VO_{2peak}$ (72.3% pred.); Elevated $AaDO_2$ in 30% and mean lactate post-exercise (5.6 mmol/l) |
| Motiejunaite et al. [72] | France | 8 | 3 months | Exertional dyspnea | 0% | NR | - | 100% and 88% incapable of reaching predicted $VO_{2max}$ and workload respectively; respiratory alkalosis and hypocapnia in 38%; elevated VE/$VCO_2$ ratio in 63%; symptom reproduction at exertion in all subjects |
| Raman et al. [73] | UK | 58 | 2.3 months (median) | Majority persistent symptoms (e.g., breathlessness , fatigue) | 100% | Symptom-limited ramp protocol on a cycle ergometer | 30 matched uninfected subjects | Reduced $VO_{2peak}$ and oxygen uptake efficiency slope; greater VE/$VCO_2$ slope compared to controls (p<0.001) |
| Rinaldo et al. [74] | Italy | 75 | 97 days (mean) | 52% with residual dyspnea | NR | Incremental, symptom-limited on a cycle ergometer | - | Reduced $VO_{2peak}$ (72% pred.) in 55% (of which 32% with HRR < 15% and 37% reduced anaerobic threshold) |
| Singh et al. [75] | USA | 10 | 11 months (mean) | Dyspnea, exercise intolerance | 10% | Invasive CPET, Ramp protocol until 85% of pred. peak HR was reached | 10 matched uninfected subjects with unexplained dyspnea | Reduced $VO_{2peak}$ (< 80% pred.) and systemic $O_2$ extraction; greater venous oxygen saturation and VE/$VCO_2$ ratio compared to controls (p<0.01) |
| Szekely et al. [76] | Israel | 71 | 91 days (mean) | 67% with persistent symptoms (e.g., fatigue, myalgia) | 4% | Symptom-limited ramp protocol on a cycle ergometer | 35 matched uninfected subjects | Lower anaerobic threshold, $O_2$ pulse and $VO_{2peak}$; higher arteriovenous oxygen difference compared to controls (p<0.05); chronotropic incompetence in 75% |

https://mc.manuscriptcentral.com/oxfimm
